# Clinical and neuropathological phenotype associated with the novel V189I mutation in the prion protein gene

**DOI:** 10.1186/s40478-018-0656-4

**Published:** 2019-01-03

**Authors:** Giuseppe Di Fede, Marcella Catania, Cristiana Atzori, Fabio Moda, Claudio Pasquali, Antonio Indaco, Marina Grisoli, Marta Zuffi, Maria Cristina Guaita, Roberto Testi, Stefano Taraglio, Maria Sessa, Graziano Gusmaroli, Mariacarmela Spinelli, Giulia Salzano, Giuseppe Legname, Roberto Tarletti, Laura Godi, Maurizio Pocchiari, Fabrizio Tagliavini, Daniele Imperiale, Giorgio Giaccone

**Affiliations:** 1 Neurology V – Neuropathology Unit, Fondazione IRCCS Istituto Neurologico Carlo Besta, Via Celoria 11, 20133 Milan, Italy; 2 Centro Regionale Malattie da Prioni (DOMP), ASL ‘Città di Torino’, Turin, Italy; 3 Neuroradiology Unit, Fondazione IRCCS Istituto Neurologico Carlo Besta, Milan, Italy; 4 Neurology Unit, Multimedica, Castellanza, Italy; 5 Neurology Unit, AO Ospedale Civile di Legnano, Legnano, Italy; 6 Neurology Unit, Foundation IRCCS Centro s. Raffaele del Monte Tabor, Milan, Italy; 7 Neurology Unit - ASST Cremona, Cremona, Italy; 8 Neurology Unit, ASL Biella, Biella, Italy; 9 Neurology Unit, Humanitas Clinical Institute Rozzano, Milan, Italy; 10 Laboratory of Prion Biology, Department of Neuroscience, Scuola Internazionale Superiore di Studi Avanzati (SISSA), Trieste, Italy; 11 Neurology Unit, Osp. Maggiore della Carità, Novara, Italy; 12 Neurology Unit, ASL Novara, Ospedale di Borgomanero, Borgomanero, Italy; 13 Department of Neuroscience, Istituto Superiore di Sanità, Rome, Italy; 14 Scientific Directorate, Fondazione IRCCS Istituto Neurologico Carlo Besta, Milan, Italy

**Keywords:** Creutzfeldt-Jakob disease, *PRNP*, Prion, V189I, CJD, PrP, Dementia, Mutation

## Abstract

Prion diseases are neurodegenerative disorders which are caused by an accumulation of the abnormal, misfolded prion protein known as scrapie prion protein (PrP^Sc^). These disorders are unique as they occur as sporadic, genetic and acquired forms. Sporadic Creutzfeldt-Jakob Disease (CJD) is the most common human prion disease, accounting for approximately 85–90% of cases, whereas autosomal dominant genetic forms, due to mutations in the prion protein gene (*PRNP*), account for 10–15% of cases. Genetic forms show a striking variability in their clinical and neuropathological picture and can sometimes mimic other neurodegenerative diseases.

We report a novel *PRNP* mutation (V189I) in four CJD patients from three unrelated pedigrees. In three patients, the clinical features were typical for CJD and the diagnosis was pathologically confirmed, while the fourth patient presented with a complex phenotype including rapidly progressive dementia, behavioral abnormalities, ataxia and extrapyramidal features, and the diagnosis was probable CJD by current criteria, on the basis of PrP^Sc^ detection in CSF by Real Time Quaking-Induced Conversion assay. In all the three patients with autopsy findings, the neuropathological analysis revealed diffuse synaptic type deposition of proteinase K-resistant prion protein (PrP^res^), and type 1 PrP^res^ was identified in the brain by western blot analysis. So, the histopathological and biochemical profile associated with the V189I mutation was indistinguishable from the MM1/MV1 subtype of sporadic CJD.

Our findings support a pathogenic role for the V189I *PRNP* variant, confirm the heterogeneity of the clinical phenotypes associated to *PRNP* mutations and highlight the importance of PrP^Sc^ detection assays as diagnostic tools to unveil prion diseases presenting with atypical phenotypes.

**Electronic supplementary material:**

The online version of this article (10.1186/s40478-018-0656-4) contains supplementary material, which is available to authorized users.

## Introduction

Prion diseases are fatal neurodegenerative disorders caused by misfolding, aggregation and accumulation of the prion protein (PrP) in brain tissue [12, 23, 39]. The pathogenic isoform of PrP (PrP^Sc^) results from a conformational change of the normal form of PrP (PrP^C^), converting α-helical regions to β-sheet motifs, that confers abnormal physicochemical properties - such as detergent insolubility and protease resistance - to PrP [8, 10], triggering its deposition in brain tissue.

**Fig. 1:**
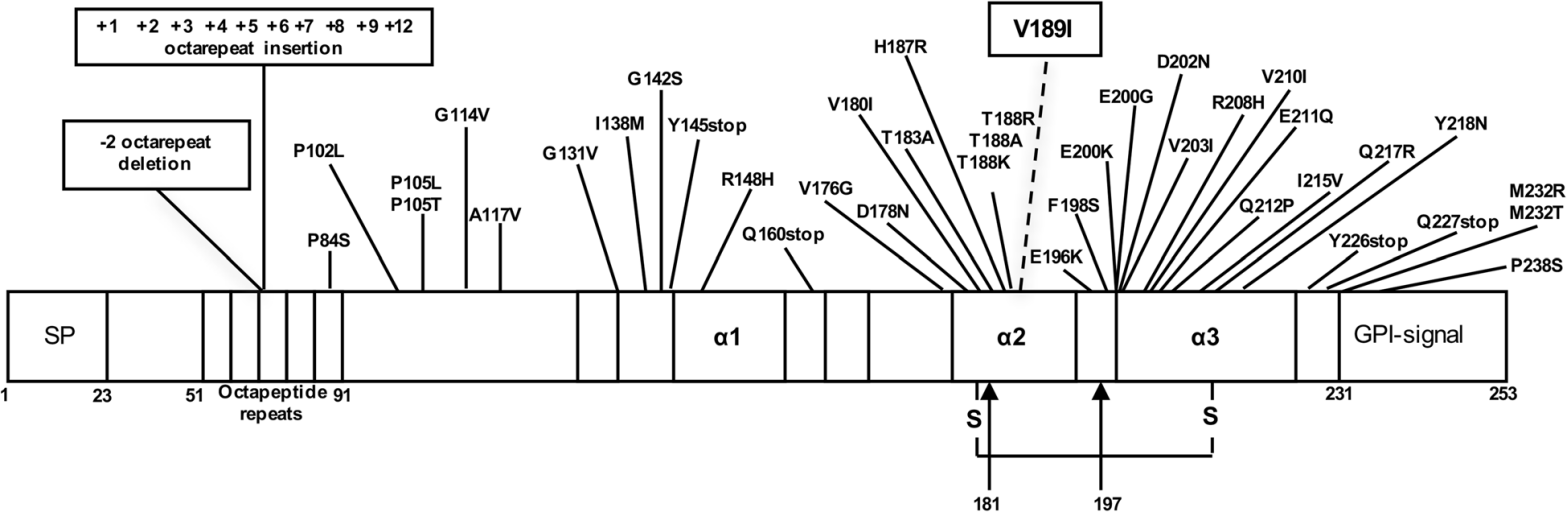
*PRNP* mutations. Single point mutations, stop-codon mutations, insertion and deletion mutations in the coding region of *PRNP* gene, which have been proposed as pathogenic variants of the prion protein. Polymorphisms or other genetic variations whose pathogenic value is unknown or uncertain are not reported in this figure

Human prion diseases include sporadic, familial and acquired forms. Distinct features separate sporadic prion diseases into three phenotypes: sporadic Creutzfeldt-Jakob disease (sCJD), sporadic fatal insomnia (sFI), and variably protease-sensitive prionopathy (VPSPr). sCJD accounts for more than 90% of all cases of sporadic prion diseases. Genetically inherited prion diseases include familial CJD (fCJD), Gerstmann-Sträussler-Scheinker disease (GSS) and fatal familial insomnia (FFI). Acquired prion diseases encompass iatrogenic CJD (iCJD), variant CJD (vCJD), and kuru [40]. vCJD occurs predominantly in the UK and has been linked to the consumption of beef products contaminated with the agent of the cattle disease, bovine spongiform encephalopathy [13, 14, 33, 40, 49]. GSS, fCJD, and FFI are caused by mutations within the open reading frame of the human prion protein gene (*PRNP)* (Fig. 1) and inherited as autosomal dominant traits. *PRNP* pathogenic mutations have been identified in 10–15% of CJD patients [37]. These mutations may be single point mutations, stop-codon mutations, or insertions or deletions of octapeptide repeats. To date, more than 50 different *PRNP* variants have been identified in large reference datasets of human genetic variations. Evidence for their pathogenic value is debated since for only a subset of these mutations convincing data coming from family history, cell culture or transgenic models are available [30]. Anyway, divergent clinicopathological phenotypes have been associated to *PRNP* mutations in several reports [6, 20, 28, 29, 36, 41, 45, 48].

Interestingly, human genetic prion diseases show inter-familial and intra-familial phenotypic variability [16, 25]. Main determinants are the type of mutation and the codon 129 genotype which affect the physicochemical properties of PrP^Sc^ and imprint disease phenotype of genetic CJD cases [35]. In some occasions, genetic factors other than *PRNP* might also influence phenotypic variability [38].

This study reports a novel *PRNP* mutation in four patients from three Italian unrelated kindred presenting with a classic CJD phenotype or an atypical clinical picture characterized by rapidly progressive dementia with behavioral changes, ataxia and extrapyramidal syndrome. Our data reinforce the view that prion diseases can occur under complex clinical phenotypes mimicking other neurodegenerative diseases and highlight the importance of employing more sensitive tools - such as PrP^Sc^ amplification assays - in the diagnostic protocols currently used to identify prionopathies.

## Materials and methods

### Clinical information

The patients underwent the clinical protocols currently used for dementias and were diagnosed according to the WHO 1998 criteria or the updated criteria by Zerr et al. [51] for CJD. The clinical pictures of the 4 cases were also interpreted based on the proposed new criteria for the diagnosis of prion diseases published on the UK CJD Surveillance website, last updated in January 2017 [27]. Neuropsychological assessment of Case 2 was performed using Milan Overall Dementia Assessment (MODA) [4]. EEGs were standard exams (duration 30–60 min) in all patients. All MRI were performed at 1.5 T scanners. The DWI sequences were performed in Cases 1 and 3. ADC maps (of Case 1 and 3) confirmed restricted diffusion in the hyperintense areas seen in DWI acquisitions. The MRI of Case 2 didn’t include DWI sequences. 14–3-3 was analyzed in CSF samples by Western blot with the pan 14–3-3 H-8 antibody (Santa Cruz Biotechnology, Santa Cruz, CA). CSF levels of total tau and phosphorylated tau were measured by ELISA (INNOTEST®, Innogenetics), according to the protocol provided by the manufacturer.

### Genetic studies

**Fig. 2:**
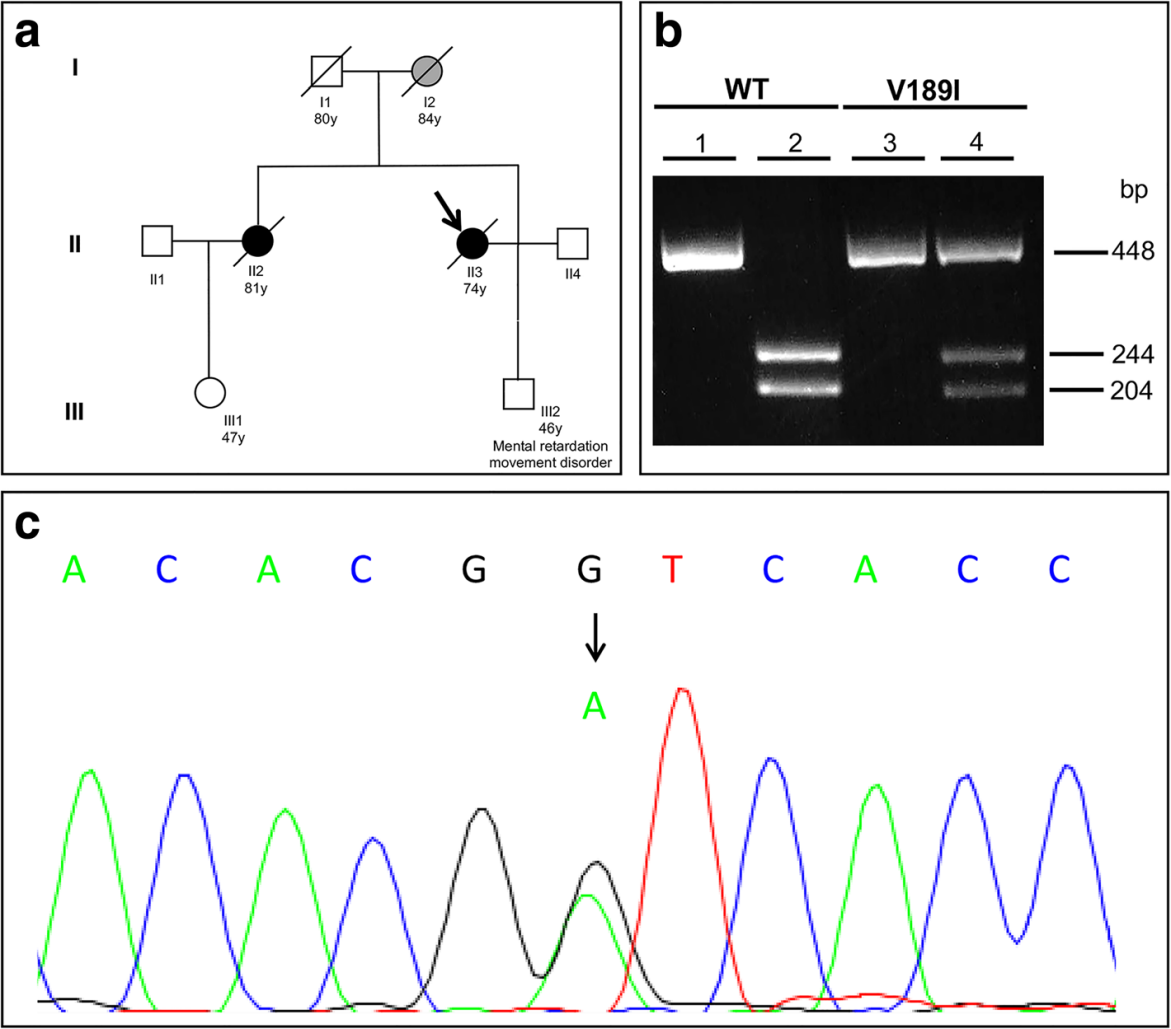
Genetic studies. **a**: Pedigree of family of Cases 1 and 2. The proband is marked by arrow, grey symbols denote family members affected by rapidly progressive dementia, black symbols indicate family members with CJD, white symbols denote unaffected members. Crossing lines refer to deceased subjects. **b**: Analysis of *PRNP* gene by restriction fragment length polymorphism. A 448 bp region was amplified by PCR from a control subject (WT, lane 1) and a mutated heterozygous carrier (V189I, lane 3) . Digestion of PCR product by BstEII generated two fragments (244 and 204 bp) in the WT subject (lane 2). The presence of the mutation abolished the restriction site. So, a 448 bp fragment (corresponding to the mutated allele) and two 244 and 204 bp fragments (corresponding to the WT allele) were observed, as expected, in the V189I heterozygous carrier (lane 4). **c**: Sequence chromatogram of a subject carrying the heterozygous V189I mutation

DNA was extracted from peripheral blood lymphocytes. Sequence analysis of full-length coding region of *PRNP*, microtubule-associated protein Tau (*MAPT*), exons 16 and 17 of amyloid-beta precursor protein (*APP*) and Presenilin 1 and 2 (*PSEN1* and *PSEN2*) genes was performed by Sanger sequencing using an ABI 3130xl DNA Analyzer (Applied Biosystems). The presence of the V189I mutation in the *PRNP* gene (Fig. 2) was confirmed by restriction fragment length polymorphism analysis; briefly, a 448 bp region was amplified by PCR using the primers 5’-CAACATGAAGCACATGGCTGGT-3′ and 5’-CCTTCCTCATCCCACTATCAGG-3′, the PCR product was digested by BstEII restriction enzyme (NEB) and resolved by electrophoresis on 2% agarose gel (Fig. 2b). We explored the recurrence of this mutation by consulting the Exome Aggregation Consortium (ExAC) database [26] and found that the V189I *PRNP* variant is not reported.

### RT-QuIC assays

Real Time Quaking-Induced Conversion (RT-QuIC) was used to detect the presence of PrP^Sc^ in cerebrospinal fluid (CSF) of patients, as previously described [11]. Briefly, 15 μl of each CSF was added to 85 μl of reaction mix in black, 96-well microplates. Samples were tested in quadruplicate together with positive (definite CJD) and negative (non-CJD) controls. The RT-QuIC reaction mix was prepared as follow: 300 mM NaCl, 10 mM phosphate buffer at pH 7.4, 1 mM ethylenediaminetetraacetic acid tetrasodium salt dehydrate (EDTA) at pH 8.0, 0.002% of Sodium dodecyl sulfate (SDS), 10 μM thioflavin-T (ThT) and 0.1 mg/ml of Syrian hamster recombinant truncated form of prion protein (Ha rPrP 90–231). After sealing, the plate was incubated in a FLUOstar OPTIMA reader (BMG Labtech) at 55 °C, over a period of 60 h with intermittent cycles of shaking (1 min) double-orbital (600 rpm) and incubation (1 min). The fluorescence intensity, expressed as arbitrary unit (AU), was taken every 15 min using 450 ± 10 nm (excitation) and 480 ± 10 nm (emission) wave-lengths. A sample was considered positive if the mean of the highest two fluorescence values (AU) of the replicates was higher than 10.000 AU and at least two out of four replicates crossed the threshold set at 60 h. The sample was considered negative if none or only one replicate (out of four) crossed the threshold before 60 h.

### Immunoblotting

Frozen samples of frontal and cerebellar cortex of patients were homogenized in phosphate buffer (pH 7.4, Sigma) at 10% (weight/volume) and were centrifuged (Eppendorf Centrifuge) at 4 °C, 800×g, for 1 min in order to remove cellular debris. Ten microliters of brain homogenates were treated with 50 μg/mL of proteinase K (PK) (Invitrogen) for 1 h at 37 °C under shaking (500 rpm). Digestion was stopped by the addition of LDS-PAGE loading buffer (Life Technologies), samples were then heated at 100 °C for 10 min and loaded into 12% Bolt Bis-Tris Plus gels (Life Technologies). Proteins were separated by means of SDS-PAGE, transferred onto Polyvinylidene difluoride (PVDF, Millipore) membrane and incubated with 5% (wt/vol) dry nonfat milk in 0.05% (vol/vol) Tween-20 (prepared in Tris-HCl) for 1 h at room temperature with shaking. PVDF membranes were finally incubated with anti-PrP antibodies 6D11 (epitopes 93–109, Covance) or 3F4 (epitopes 109–112, Dako) and developed with chemiluminescent system (ECL Prime, GE Healthcare Amersham).

### Neuropathology

The neuropathological study was carried out on formalin-fixed sections of the cerebral hemispheres and cerebellum. Ten-micrometer-thick sections of several brain areas including frontal, temporal, parietal and occipital cortex, hippocampus, striatum and thalamus were stained with hematoxylin-eosin (H&E), cresyl violet for Nissl substance and thioflavin S for amyloid or immunolabeled for PrP^Sc^ using the mouse monoclonal antibody 3F4 (1:200; epitope at residues 109–112 of human PrP, DakoCytomation). Before overnight incubation with 3F4 antibody, the sections were pretreated with autoclaving in distilled water (121 °C, 10 min), followed by exposure to formic acid (98%, 5 min) and by guanidine thiocyanate (3 M, 20 min), as previously described [15]. Additional sections were immunostained with a polyclonal anti-glial fibrillary acidic protein (GFAP) antibody (DakoCytomation, 1:1000). EnVision (DakoCytomation) was used as detection system and 3–3′-diaminobenzidine (DAB) as chromogen.

## Results

### Case reports

#### Case 1

She was a 74-year-old woman (Table 1), whose family history revealed that her mother complained of dementia and visual hallucinations with onset at 83 years and died at the age of 84 years. The disease duration was 8 months. The proband’s sister suffered of a dementing illness whose phenotype is described as Case 2 in this paper. A 46-year-old son of the proband was affected by mental retardation and movement abnormalities probably caused by a congenital malformation mainly involving cerebellum (Fig. 2a).

**Table 1:** Clinical, neuropathological and biochemical features of the V189I carriers

	Case 1	Case 2	Case 3	Case 4
Family history for CJD	Yes	Yes	No	No
Gender	Female	Female	Male	Male
Age at onset	74 yrs	78 yrs	71 yrs	69 yrs
Disease duration	5 mo	33 mo	4 mo	4 mo
Symptoms at onset	Visual hallucinations, abnormal behavior	Ataxia, cognitive impairment	Short-term memory deficits, fluctuating confusion, depression	Ataxia, writing difficulties and behavior changes
Myoclonus	–	–	+	+
Other neurological findings	Speech impairment and asymmetric pyramidal signs	Extrapyramidal syndrome, visual hallucinations, abnormal behavior	Ataxia, cerebellar deficits	Cerebellar deficits
EEG	Background delta rhythm and recurrent theta sharp waves	Diffuse slowing of the background activity	Inconstant bilateral periodic sharp wave complexes	Theta-delta activity in fronto-temporal regions without PSWs
MRI	High signal in caudate heads and diffuse hyperintensity in the cortex in DWI images	Diffuse cortical atrophy mainly involving left frontal and temporal lobi	Hyperintensity in DWI images in frontal and parietal right cortex and in right cingulus	Hyperintensity in DWI sequences in bilateral fronto-parietal and left insular cortices and in the right thalamus
CSF analysis	14–3-3 positive	14–3-3 negative	14–3-3 positive	14–3-3 weakly positive
	Tau 3433 pg/ml	Tau 392 pg/ml	Tau 9250 pg/ml	Tau 1780 pg/ml
CSF RT-QuIC assay	+	+	+	n/a
M/V polymorphism at 129 PRNP codon	M/M	M/V	M/M	M/M
Histological and immunohistochemical findings	Diffuse spongiosis, cell loss and gliosis; diffuse, finely granular, synaptic-type PrP immunoreactivity	n/a	Diffuse spongiform changes; faint synaptic deposition in the cerebrum, molecular layer of the cerebellum, thalamus and striatum	Diffuse microspongiosis with relative sparing of hippocampus and brainstem; faint synaptic PrP^Sc^ deposition
PrP type	Type 1	n/a	Type 1	Type 1

The proband’s disease began two months before her admission to hospital with visual hallucinations, delusions, overvalued ideas and confabulation, rapidly evolving towards confusion, psychomotor slowness, abnormal behavior, loss of autonomy in daily life activities and incontinence. Serial CT brain scans during this period showed only a mild atrophy in frontal lobes.

During the last week before hospitalization, the clinical picture was characterized by fast psychomotor deterioration. The patient became unable to walk and showed clear speech difficulties, tonic grasping, asymmetric hypertonia involving mainly left arms, reduced alertness.

**Fig. 3:**
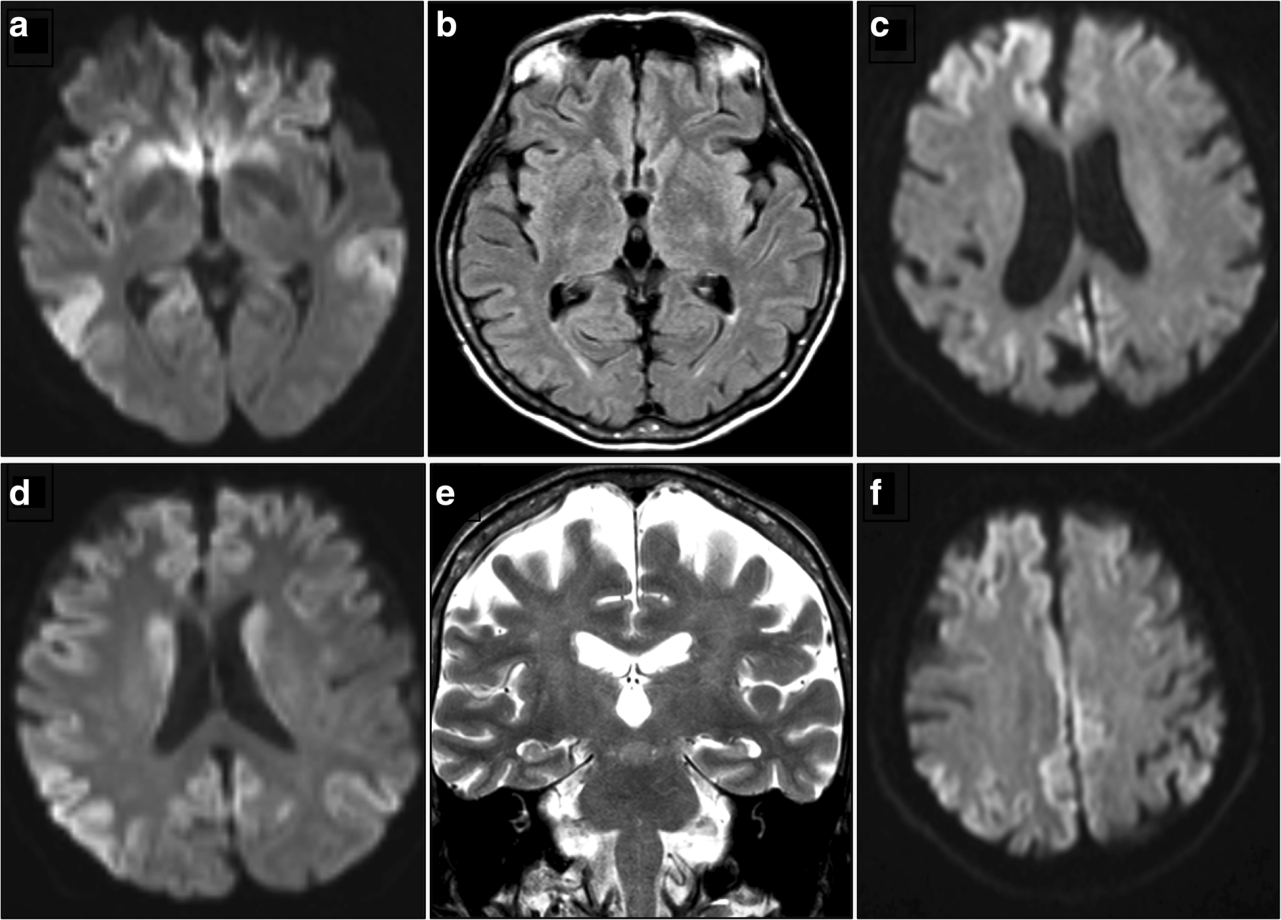
Imaging studies. **a, d**: DWI images show diffuse signal abnormalities involving bilaterally the posterior temporal cortex, caudate and putamen, parietal and frontal cortex, more marked in the right side (Case 1). **b**, **e**: Axial Flair and coronal T2-weighted images show diffuse cortical atrophy, involving frontal lobe with mild left prevalence (Case 2). **c**, **f**: DWI images show marked signal abnormalities in frontal and parietal right cortex and in right cingulus (Case 3)

Electroencephalogram (EEG) showed a slow background activity (delta rhythm) and the presence of recurrent theta sharp waves especially in the anterior brain regions. No periodic wave complexes were observed in two different EEG recordings performed 3 months after the onset of the disease, during the hospitalization. Brain DWI MR images (Fig. 3, panels a,d) showed high signal in caudate heads and diffuse hyperintensity in the cortex with predominance of frontal and parietal lobes; cortical atrophy of frontal lobes; mild leukoaraiosis.

CSF analysis showed the presence of 14–3-3 protein. Total tau and phosphorylated tau levels in CSF were 3433 pg/ml (n.v. < 500 pg/ml) and 44 pg/ml (n.v. < 61 pg/ml), respectively.

She died five months after the onset of the disease and underwent autopsy. Her neuropathological picture is detailed below (see *Neuropathology* paragraph).

**Fig. 4:**
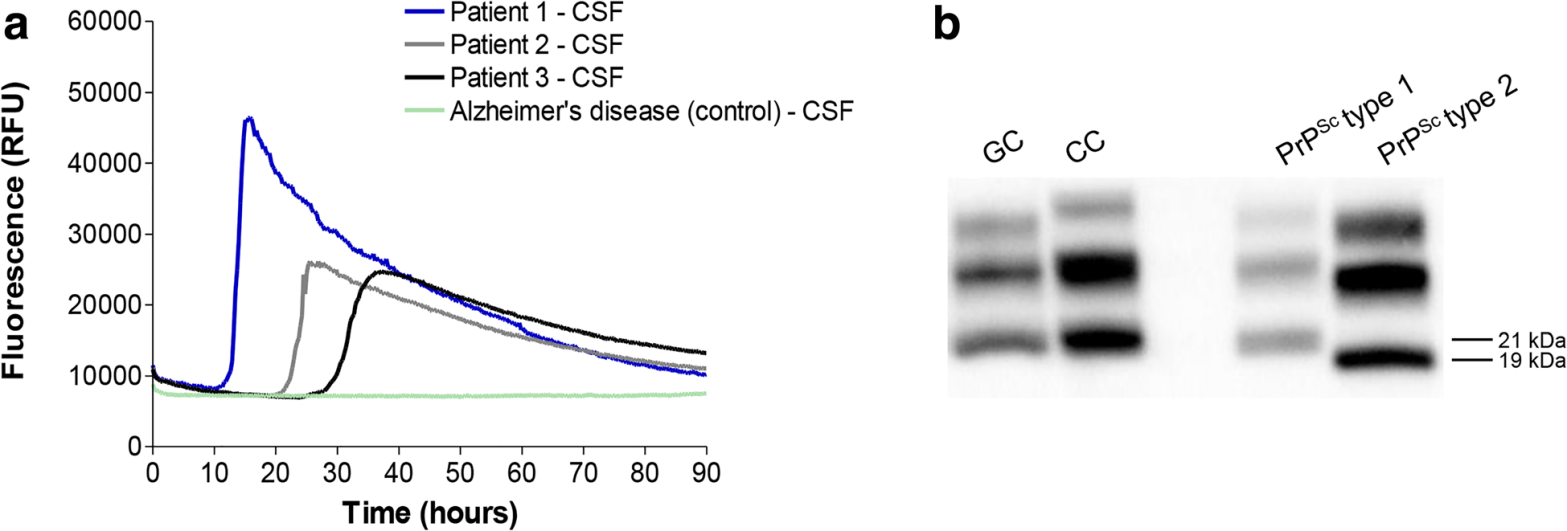
Biochemical studies. **a** RT-QuIC analysis: 15 μL of CSF collected from patients 1, 2 and 3 efficiently seeded the aggregation of recHaPrP (90–231) while CSF collected from patient with AD (referred to as control) did not. The mean ThT fluorescence values per sample were plotted against time. **b** Western blot analysis: frozen samples of frontal (GC) and cerebellar (CC) cortex from Case 1 showed the presence of type 1 PrP^Sc^ after digestion with PK (50 μg/mL). Frontal cortex of patients with type 1 and type 2 PrP^Sc^ were used as migration controls. Numbers on the right side of the WB indicate the molecular weight

The CSF study was completed after death by amplification PrP^Sc^ assay with RT-QuIC. The test was positive, confirming the presence of pathological prion protein in CSF sample of the patient (Fig. 4a).

#### Case 2

The patient was the 80-year-old sister of the Case 1 (Fig. 2). She had blood hypertension and hyperthyroidism since the age of 53 years. She was referred to the hospital for a 2-year history of progressive gait imbalance with recurrent falls, mild cognitive decline and depression with weight loss (Table 1). A brain CT scan showed leukoaraiosis and diffuse cerebral atrophy.

On admission, the patient was unable to stand and walk due to ataxia, extrapyramidal syndrome and mild hyposthenia of legs. No relevant pyramidal signs were observed. Impairment of cerebellar function and multifocal cognitive loss was noticed by neurologic examination. Behavioral abnormalities with delusions, visual hallucinations, confabulations, mental confusion were evident in the following days. These symptoms were scarcely responsive to Quetiapine but were partially controlled by the use of Haloperidol.

Neuropsychological assessment showed behavioral disturbances with depression and visual hallucinations, moderate-to-severe cognitive dysfunctions, mainly consisting of impairment of thought content and semantic fluency, bad orientation in time and place, memory deficits, confabulation, dyspraxia.

Laboratory analysis revealed increase of FT4 (9.9 ng/dL, n.v. 0,70-1,48) and low TSH levels (0.13 μUI/mL, n.v. 0,45-3,50). The imbalance of thyroid function was corrected by the adjustment of pharmacologic treatment for hyperthyroidism with transient positive effects on psychic disturbances (regression of delusions/hallucinations and improvement of mental confusion). CSF analysis showed absence of 14–3-3 protein. CSF levels of total tau protein were 392 pg/ml (n.v. < 500 pg/ml). EEG showed a diffuse slowing of the background activity towards the delta rhythm. An improvement of the EEG profile was observed after neuroleptic treatment and correction of thyroid dysfunction. Brain MRI showed multiple little ischemic foci in white matter of both brain hemispheres, and diffuse cortical atrophy involving mainly left frontal and temporal lobi (Fig. 3, panels b,e). All these clinical investigations were performed 2 years after the disease onset.

Due to the prominence of behavioral changes in her clinical picture and to brain MRI findings, the patient was initially diagnosed as frontotemporal dementia (FTD).

Over the following months, the clinical picture evolved towards tetraparesis, severe ataxia, and further cognitive deterioration. When the family history of the patient emerged and the presence of a *PRNP* mutation was confirmed in her sister (Case 1), the analysis of *PRNP* gene was carried out also in this patient and revealed the presence of the Valine-to-Isoleucine substitution at codon 189 with Methionine/Valine polymorphism at codon 129.

The patient died nine months after hospital discharge, around 33 months after the onset of the disease. No autopsy was performed. However, RT-QuIC analysis of CSF sample collected *in vitam* was carried out and was positive, confirming the presence of pathological prion protein.

Taking into account her clinical findings, Case 2 met the WHO 1998 criteria or the updated criteria by Zerr et al. [51] for ‘possible’ sCJD, as she had no positive ancillary tests. However, the results of the RT-QuIC test on CSF, made reasonable a diagnosis of ‘probable’ CJD according to the proposed new criteria from the UK and Germany that allow any neurological syndrome with a positive RT-QuIC.

#### Case 3

This patient was a 71-year-old man with a 2-month history of short-term memory deficits and fluctuating confusion (Table 1). The family history was unremarkable except for two cases of late-onset depression (> 60 years) in two sisters of his father. The patient underwent neurologic evaluation that resulted to be normal: a presumptive diagnosis of reactive depression was made and a treatment with sertraline was suggested. Since the lack of response and the worsening of cognitive symptoms, the patient was subjected to a brain MRI study that showed marked signal abnormalities in frontal and parietal right cortex and in right cingulum in DWI sequences (Fig. 3, panels c,f). A further neurologic examination disclosed a mild temporal disorientation with bilateral cerebellar dysmetria with dysdiadochokinesia and gait unbalance. Moreover, rare myoclonic jerks were evident.

The EEG pattern was possibly suggestive of a prion disease since the inconstant occurrence of bilateral periodic sharp wave complexes. CSF analysis showed the presence of 14–3-3 protein. Total tau in CSF was 9250 pg/ml (n.v. < 500 pg/ml) and phosphorylated tau 42 pg/ml (n.v. < 61 pg/ml). RT-QuIC analysis of CSF sample was positive.

Overall these tests were performed 2,5 months after disease onset.

A diagnosis of probable CJD was made.

The clinical picture rapidly deteriorated and the patient became tetraparetic, and unable to speak and swallow in two weeks. Therefore, he was transferred to his community hospital in the North-East of Piemonte where he died 2 months from the first hospital admission due to a multi-organ failure. Autopsy was performed to confirm CJD diagnosis.

#### Case 4

This patient was a 69-year-old man with no family history of dementia or neurodegenerative diseases (Fig. 2), who was admitted to his community hospital because of progressive gait unbalance, writing difficulties and behavior changes started in the previous two months (Table 1). On neurologic examination, cerebellar ataxia with dysmetria and dysdiadochokinesia were evident as well as spatiotemporal disorientation. MMSE score was 12/30.

On brain MRI, there was an hyperintensity in DWI sequences at level of bilateral frontoparietal and left insular cortices and, mildly, at level of the right posterior thalamic region with no Gadolinium enhancement. All the EEG recordings were not typical for a prion disease, being characterized by a bilateral theta-delta activity in frontotemporal regions without evidence of PSWs. CSF analysis showed a weak 14–3-3 positivity with total tau levels of 1780 pg/ml and phosphorylated tau of 73.4 pg/ml, respectively. MRI, EEG, and CSF analysis were performed 2 months after the onset of the symptoms.

A diagnosis of probable CJD was made.

Clinical picture rapidly evolved towards a persistent vegetative status with diffuse spontaneous myoclonus. The patient died two months after the hospital admission because of a multi-organ failure and underwent autopsy to confirm CJD diagnosis.

### Genetic studies

The genetic analysis revealed the unprecedented Valine-to-Isoleucine substitution at codon 189 in all the 4 patients (Fig. 2, panels b and c) and the Methionine/Methionine polymorphism at codon 129 in Cases 1, 3 and 4, while the genotype at codon 129 of the Case 2 was Methionine/Valine (Table 1).

### RT-QuIC assays

RT-QuIC performed on CSF samples from Cases 1, 2 and 3 was positive, indicating the presence of PrP^Sc^ (Fig. 4, panel a). CSF from Case 4 was not available for RT-QuIC analysis. CSF collected from a patient with Alzheimer’s disease (AD) was used as negative control.

### Immunoblotting

Western blot analysis of both gyrus cinguli and cerebellar cortex of Case 1 revealed the presence of typical type 1 PrP^Sc^ with the unglycosylated band migrating at 21 kDa (Fig. 4, panel b). Gyrus cinguli of patients with sCJD with type 1 and type 2 PrP^Sc^ were used as migration controls. All samples were treated with proteinase K (50 μg/mL) before analysis and immunoblotted with the 3F4 antibody. The same findings were obtained by immunoblot analysis on the brain samples of Cases 3 and 4.

### Neuropathology

#### Cases 1, 3 and 4

**Fig. 5:**
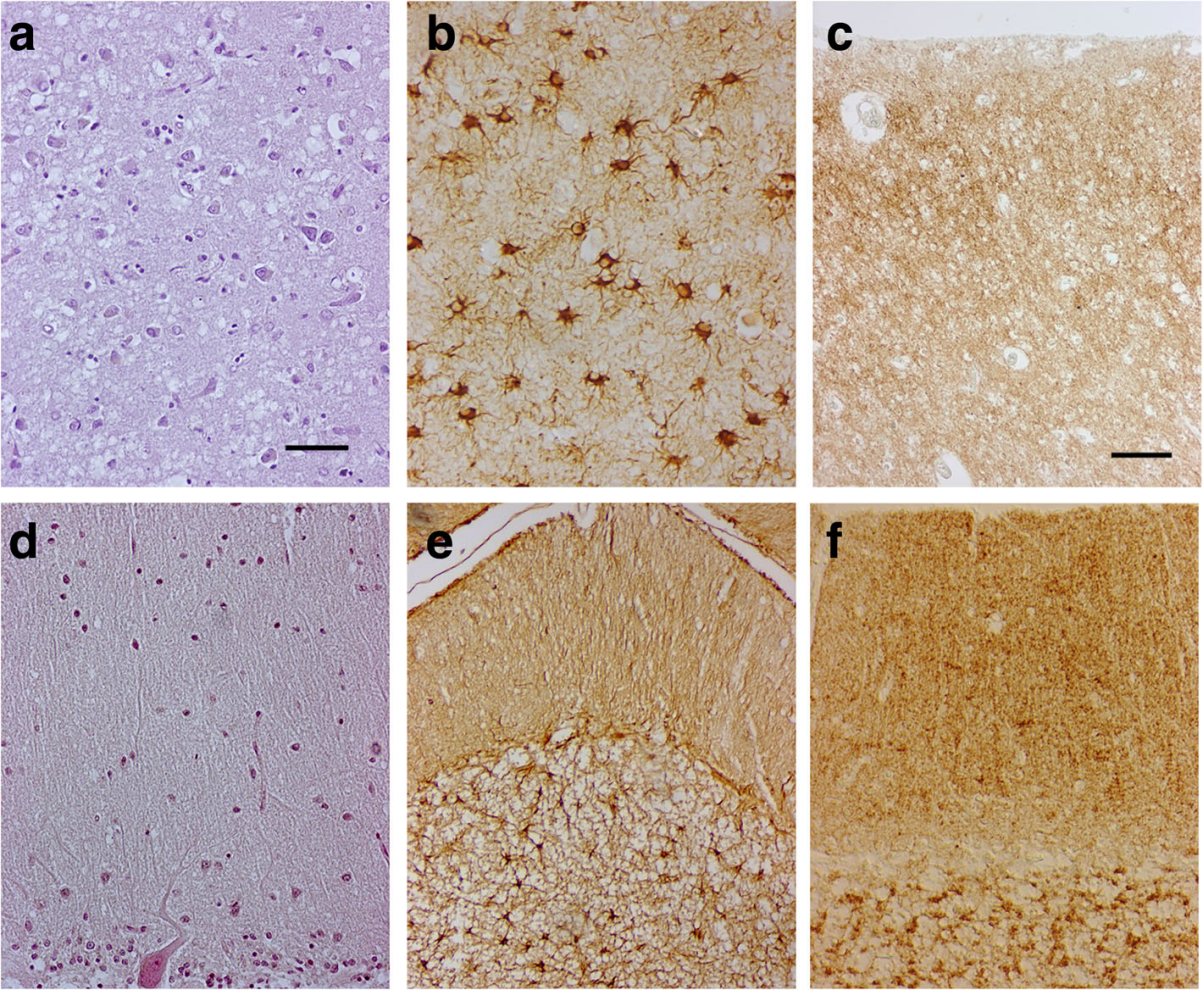
Neuropathology of Case 1. The neuropathological analysis showed the presence of severe neuronal loss and spongiform changes in the cerebral cortex (**a**: frontal cortex, Haematoxylin-Eosin), associated with astrogliosis (**b**: frontal cortex, GFAP immunostaining). The pattern of PrP^Sc^ deposition was defined by diffuse, finely granular synaptic-like immunoreactivity (**c**: 3F4 immunostaining, frontal cortex). In the cerebellum, loss of Purkinje and very mild spongiosis in the molecular layer (**d**: Haematoxylin-Eosin), astrogliosis (**e**: GFAP immunostaining) and PrP build up were present: finely granular PrP deposits in the molecular layer and coarser spots in the granular layer (**f**: 3F4 immunostaining). The PrP deposits were not fluorescent after thioflavin S (not shown). Scale bars: in (**a**) = 100 μm (**a**, **b**, **d** and **f** are the same magnification); in (**c**) = 50 μm (**c** and **e** are the same magnification).

The neuropathological and PrP immunohistochemical patterns of the three patients were very similar and closely corresponded to the MM/V1 histotype of CJD by Parchi [33]. The neuropathological examination revealed spongiosis, nerve cell loss and gliosis associated with PrP^Sc^ immunoreactivity (Fig. 5 and Additional file 1: Figure S1). Moderate to severe spongiform changes were observed in all the areas of the cerebral cortex examined and in the striatum. Diffuse, finely granular, “synaptic-type” PrP immunoreactivity homogeneously involved the cerebral cortex, striatum, thalamus. No large, coalescent cortical vacuoles of spongiosis associated with perivacuolar PrP^Sc^ immunoreactivity were detected. The cerebellum showed moderate Purkinje and granule cell loss, mild spongiosis in the molecular layer and focal areas of PrP^Sc^ immunoreactivity as fine-dotted staining in the molecular layer and a coarse-dotted staining in the granular layer. PrP amyloid deposits were not present.

## Discussion

We have discovered a novel mutation in the *PRNP* gene (V189I) in four patients affected from CJD. In 3 out of 4 cases the V189I *PRNP* variant was associated with a clinicopathological phenotype and a biochemical profile indistinguishable from the MM1 subtype of sporadic CJD previously described [5, 13, 34]. In these 3 patients, the course of the disease was fast with rapid neurological deterioration and death occurring few months after onset, indicating a severe pathogenic effect of the mutation. Only in one V189I carrier (reported as Case 2 in this paper), the clinical presentation of the disease was milder and the duration of the illness longer, so the diagnosis of CJD was made only when the family history of the patient emerged and the presence of a *PRNP* mutation was confirmed in her sister (Case 1). The clinical data were then revised and the RT-QuIC was performed in the CSF with a positive result, supporting the diagnosis of CJD.

In our view, a pathogenic role of the V189I mutation is supported by its identification in three pathologically confirmed CJD patients and in a fourth case likely developing a milder form of CJD. Moreover, the V189I *PRNP* variant was not found in the ExAc database that includes more than 60,000 human genomes.

The Valine residue at codon 189 of *PRNP* was reported to be highly conserved throughout mammalian organisms, suggesting that a mutation occurring at this site of the gene may have relevant effects on PrP function [44]. Previous studies indicated that mutations in the PrP segment containing α2-helix – e.g. mutations at 188 codon - may have β-sheet promoting effects and may result in structural destabilization of the protein [42]. α2-helix is characterized by a strong propensity for the extended conformation, and a single amino acid replacement within or in proximity with this helix is shown to significantly affect the conformational preference of the entire α2-helix/α3-helix segment and to facilitate conformational rearrangement in this region promoting the extended conformation of this domain [21, 24]. These findings also correlate with the high number of pathogenic mutations in α2 and α3 helices, which emphasize the relevance of these helices for conformational transitions. In fact, only one disease-promoting mutation was found in α1-helix while at least seven and ten such mutations were found in α2- and α3 helices respectively [2, 19]. A previous in silico study by Kedarisetti et al. [19] indicated the V189I substitution - together with several other amino acid changes including V210I, P137M, G142D, G142 N, D144P, K185 T, H187Y and T191P - as a variation in the sequence of the protein that may potentially affect structural stability. We investigated on the potential effects of the Valine-to-Isoleucine substitution at *PRNP* codon 189 also by interrogating several prediction analysis software - including SIFT (https://sift.bii.a-star.edu.sg) [46], PolyPhen2 (http://genetics.bwh.harvard.edu/pph2/) [1], MAPP (http://mendel.stanford.edu/SidowLab/downloads/MAPP/index.html) [47], PREDICTSNP (https://loschmidt.chemi.muni.cz/predictsnp/) [3], MUpro (http://mupro.proteomics.ics.uci.edu/) [7] - among others - and obtained conflicting findings, since some results suggested a tolerated variation and others supported the pathogenicity of the V189I mutation. On these bases, we are not sure that the pathogenic outcomes of the V189I variant depend on the same effects on the protein structure reported for other mutations in the α2-helix of prion protein. Other unknown mechanisms may be involved.

Interestingly, an increasing number of *PRNP* mutations have been recently linked to neurodegenerative diseases other than CJD, including FTD-like [18, 31], AD-like [2, 52] clinical pictures or other unique clinical phenotypes [2]. Since many of these mutations were not linked to a detailed neuropathological study [2], it is possible that at least a part of them is associated to prion diseases *clinically* mimicking other forms of dementias, like it happened in one of our V189I carriers (Case 2).

The longer disease duration and the divergent clinical phenotype of the Case 2 could be ascribed to the well-known effects of the M/V polymorphism at codon 129 of *PRNP* [32, 43, 50]. Indeed, M/V-129 genotype is generally associated with milder CJD phenotypes and M/V polymorphism was reported to modulate the onset and severity of the disease even in genetically inherited forms of CJD [6, 22, 28, 33].

Our data confirm that neuropathological findings in genetic CJD may be indistinguishable from those of the MM/MV1 histotype, the most common found in sporadic CJD. This is also the case of the previously reported CJD patients who harbor different *PRNP* mutations in the adjacent codon 188 [42], while mutation H187R of *PRNP* is associated with GSS phenotype [9, 17]. Moreover, the present report reinforces the concept that distinct phenotypes may occur in association with the same *PRNP* mutation also within the same family.

The absence of remarkable family history in the Cases 3 and 4 may suggest a low penetrance of the V189I mutation, similarly to other Valine-to-Isoleucine substitutions in the *PRNP* gene, like V180I and V210I [6, 45]. Further studies enrolling other V189I carriers should be carried out to confirm this interpretation.

Considering the absence of neuropathological studies supporting the hypothesis of a prion disease in Case 2, and the limited clinical data available for this patient, we cannot rule out that Case 2 developed a different disease. However, the rapid progression of her dementia, the presence of PrP^Sc^ in her CSF and the existence of two other family members with a rapidly progressive dementia, one of them with a neuropathologically confirmed diagnosis of CJD, may support the diagnosis of ‘probable’ CJD according to the proposed new criteria for prion diseases [44] or at least ‘possible’ CJD according to the WHO 1998 criteria or the updated criteria by Zerr et al. [51].

Lack of distinctive clinical and pathological features in many genetic forms of prion diseases, possible presentation with clinical pictures not typical for CJD and absence of familiar history due to penetrance lower than 100% suggest that the routine sequencing of *PRNP* gene in CJD surveillance is necessary to provide a correct identification of sporadic and genetic prion diseases.

## Conclusions

We report a novel *PRNP* mutation (V189I) whose hystopathological and biochemical profiles closely match those associated with the MM1/MV1 subtype of sCJD. Our findings support a pathogenic role for the V189I *PRNP* variant and provide further suggestions on the heterogeneity of the clinical phenotypes associated to *PRNP* mutations. Additional data coming from studies in larger cohorts of V189I carriers, however, are requested to confirm that the V189I variant can generate different CJD phenotypes.

Finally, our data further stress the relevance of PrP^Sc^ detection assays as powerful tools in diagnostic protocols for prion encephalopathies, especially for the recognition of atypical phenotypes of prion diseases.

## Additional file

Additional file 1: **Figure S1.** Neuropathology of case 3. The neuropathological study on brain sample from case 3 showed findings overlapping those found in case 1 and 4. Severe neuronal loss and spongiform changes (**a**: frontal cortex, Haematoxylin-Eosin) associated with astrogliosis (**b**: frontal cortex, GFAP immunostaining) were observed in cerebral cortex. The pattern of PrP^Sc^ deposition was the same of cases 1 and 4, and consisted of diffuse, synaptic-like immunoreactivity (**c**: 3F4 immunostaining, frontal cortex). Similar changes were found in cerebellum: loss of Purkinje cells and spongiosis in the molecular layer (**d**: Haematoxylin-Eosin), diffuse astrogliosis in the granular layer (**e**: GFAP immunostaining) and finely granular PrP deposits in the molecular layer (**f**: 3F4 immunostaining). As in the other cases carrying the V189I mutation, coarse spots of PrP immunostaining were evident in the granular layer (**f**: 3F4 immunostaining). Scale bars: in (**a**) = 100 μm (**a**, **b**, **d** and **f** are the same magnification); in (**c**) = 50 μm (**c** and **e** are the same magnification). (PDF 2218 kb)

## Abbreviations

*APP*: Amyloid-beta Precursor Protein
CJD: Creutzfeldt-Jakob Disease
fCJD: Familial Creutzfeldt-Jakob Disease
FFI: Fatal Familial Insomnia
FTD: Frontotemporal Dementia
GSS: Gerstmann-Sträussler-Scheinker disease
iCJD: Iatrogenic Creutzfeldt-Jakob Disease
*MAPT*: Microtubule Associated Tau Protein
MMSE: Mini-Mental State Examination
*PRNP*: Prion Protein Gene
PrP: Prion Protein
PrP^C^: Cellular Prion Protein
PrP^res^: Prion Protein resistant to degradation by endogenous proteases
PrP^Sc^: Scrapie-associated Prion Protein
*PSEN1*: Presenilin 1 gene
*PSEN2*: Presenilin 2 gene
recHaPrP: recombinant Syrian hamster truncated form of prion protein
RT-QuIC: Real Time Quaking-Induced Conversion
sCJD: Sporadic Creutzfeldt-Jakob Disease
vCJD: Variant Creutzfeldt-Jakob Disease

## References

[1] Adzhubei IA, Schmidt S, Peshkin L, Ramensky VE, Gerasimova A, Bork P, Kondrashov AS, Sunyaev SR (2010). A method and server for predicting damaging missense mutations. Nat Methods.

[2] Bagyinszky E, Giau VV, Youn YC, An SSA, Kim S (2018). Characterization of mutations in PRNP (prion) gene and their possible roles in neurodegenerative diseases. Neuropsychiatr Dis Treat, City.

[3] Bendl J, Stourac J, Salanda O, Pavelka A, Wieben ED, Zendulka J, Brezovsky J, Damborsky J (2014). PredictSNP: robust and accurate consensus classifier for prediction of disease-related mutations. PLoS Comput Biol.

[4] Brazzelli M, Capitani E, Della Sala S, Spinnler H, Zuffi M (1994). A neuropsychological instrument adding to the description of patients with suspected cortical dementia: the Milan overall dementia assessment. J Neurol Neurosurg Psychiatry.

[5] Cali I, Castellani R, Yuan J, Al-Shekhlee A, Cohen ML, Xiao X, Moleres FJ, Parchi P, Zou WQ, Gambetti P (2006). Classification of sporadic Creutzfeldt-Jakob disease revisited. Brain.

[6] Capellari S, Strammiello R, Saverioni D, Kretzschmar H, Parchi P (2011). Genetic Creutzfeldt-Jakob disease and fatal familial insomnia: insights into phenotypic variability and disease pathogenesis. Acta Neuropathol.

[7] Cheng J, Randall A, Baldi P (2006). Prediction of protein stability changes for single-site mutations using support vector machines. Proteins.

[8] Collinge J, Clarke AR (2007). A general model of prion strains and their pathogenicity. Science.

[9] Colucci M, Moleres FJ, Xie ZL, Ray-Chaudhury A, Gutti S, Butefisch CM, Cervenakova L, Wang W, Goldfarb LG, Kong Q (2006). Gerstmann-Straussler-Scheinker: a new phenotype with ‘curly’ PrP deposits. J Neuropathol Exp Neurol.

[10] Di Fede G, Giaccone G, Salmona M, Tagliavini F (2017) Translational research in Alzheimer's and prion diseases. J Alzheimers Dis: Doi. 10.3233/JAD-170770 10.3233/JAD-170770 PMC5869996 29172000

[11] Franceschini A, Baiardi S, Hughson AG, McKenzie N, Moda F, Rossi M, Capellari S, Green A, Giaccone G, Caughey B (2017). High diagnostic value of second generation CSF RT-QuIC across the wide spectrum of CJD prions. Sci Rep.

[12] Gambetti P, Cali I, Notari S, Kong Q, Zou WQ, Surewicz WK (2011). Molecular biology and pathology of prion strains in sporadic human prion diseases. Acta Neuropathol.

[13] Gambetti P, Kong Q, Zou W, Parchi P, Chen SG (2003). Sporadic and familial CJD: classification and characterisation. Br Med Bull.

[14] Ghetti B, Tagliavini F, Takao M, Bugiani O, Piccardo P (2003). Hereditary prion protein amyloidoses. Clin Lab Med.

[15] Giaccone G, Canciani B, Puoti G, Rossi G, Goffredo D, Iussich S, Fociani P, Tagliavini F, Bugiani O (2000). Creutzfeldt-Jakob disease: Carnoy's fixative improves the immunohistochemistry of the proteinase K-resistant prion protein. Brain Pathol.

[16] Giovagnoli AR, Di Fede G, Aresi A, Reati F, Rossi G, Tagliavini F (2008). Atypical frontotemporal dementia as a new clinical phenotype of Gerstmann-Straussler-Scheinker disease with the PrP-P102L mutation. Description of a previously unreported Italian family. Neurol Sci.

[17] Hall DA, Leehey MA, Filley CM, Steinbart E, Montine T, Schellenberg GD, Bosque P, Nixon R, Bird T (2005). PRNP H187R mutation associated with neuropsychiatric disorders in childhood and dementia. Neurology.

[18] Jansen C, Parchi P, Capellari S, Vermeij AJ, Corrado P, Baas F, Strammiello R, van Gool WA, van Swieten JC, Rozemuller AJ (2010). Prion protein amyloidosis with divergent phenotype associated with two novel nonsense mutations in PRNP. Acta Neuropathol.

[19] Kedarisetti KD, Dick S, Kurgan L (2008). Searching for factors that distinguish disease-prone and disease-resistant prions via sequence analysis. Bioinform Biol Insights.

[20] Kim MO, Cali I, Oehler A, Fong JC, Wong K, See T, Katz JS, Gambetti P, Bettcher BM, Dearmond SJ (2013). Genetic CJD with a novel E200G mutation in the prion protein gene and comparison with E200K mutation cases. Acta Neuropathol Commun.

[21] Knaus KJ, Morillas M, Swietnicki W, Malone M, Surewicz WK, Yee VC (2001). Crystal structure of the human prion protein reveals a mechanism for oligomerization. Nat Struct Biol.

[22] Kobayashi A, Teruya K, Matsuura Y, Shirai T, Nakamura Y, Yamada M, Mizusawa H, Mohri S, Kitamoto T (2015). The influence of PRNP polymorphisms on human prion disease susceptibility: an update. Acta Neuropathol.

[23] Kovacs GG, Budka H (2009). Molecular pathology of human prion diseases. Int J Mol Sci.

[24] Kuznetsov IB, Rackovsky S (2004). Comparative computational analysis of prion proteins reveals two fragments with unusual structural properties and a pattern of increase in hydrophobicity associated with disease-promoting mutations. Protein Sci.

[25] Ladogana A, Kovacs GG (2018). Genetic Creutzfeldt-Jakob disease. Handb Clin Neurol.

[26] Lek M, Karczewski KJ, Minikel EV, Samocha KE, Banks E, Fennell T, O’Donnell-Luria AH, Ware JS, Hill AJ, Cummings BB (2016). Analysis of protein-coding genetic variation in 60,706 humans. Nature.

[27] Mackenzie G, Will R (2017). Creutzfeldt-Jakob disease: recent developments. F1000Res.

[28] Mancuso M, Siciliano G, Capellari S, Orsucci D, Moretti P, Di Fede G, Suardi S, Strammiello R, Parchi P, Tagliavini F (2009). Creutzfeldt-Jakob disease with E200K PRNP mutation: a case report and revision of the literature. Neurol Sci.

[29] Mauro C, Giaccone G, Piscosquito G, Lavorgna A, Nigro M, Di Fede G, Leonardi A, Coppola C, Formisano S, Tagliavini F (2008). A novel insertional mutation in the prion protein gene: clinical and bio-molecular findings. J Neurol Neurosurg Psychiatry.

[30] Minikel EV, Vallabh SM, Lek M, Estrada K, Samocha KE, Sathirapongsasuti JF, McLean CY, Tung JY, Yu LP, Gambetti P (2016). Quantifying prion disease penetrance using large population control cohorts. Sci Transl Med.

[31] Oldoni E, Fumagalli GG, Serpente M, Fenoglio C, Scarioni M, Arighi A, Bruno G, Talarico G, Confaloni A, Piscopo P (2016). PRNP P39L variant is a rare cause of frontotemporal dementia in Italian population. J Alzheimers Dis.

[32] Palmer MS, Dryden AJ, Hughes JT, Collinge J (1991). Homozygous prion protein genotype predisposes to sporadic Creutzfeldt-Jakob disease. Nature.

[33] Parchi P, de Boni L, Saverioni D, Cohen ML, Ferrer I, Gambetti P, Gelpi E, Giaccone G, Hauw JJ, Hoftberger R (2012). Consensus classification of human prion disease histotypes allows reliable identification of molecular subtypes: an inter-rater study among surveillance centres in Europe and USA. Acta Neuropathol.

[34] Parchi P, Giese A, Capellari S, Brown P, Schulz-Schaeffer W, Windl O, Zerr I, Budka H, Kopp N, al PP (1999). Classification of sporadic Creutzfeldt-Jakob disease based on molecular and phenotypic analysis of 300 subjects. Ann Neurol.

[35] Petersen RB, Goldfarb LG, Tabaton M, Brown P, Monari L, Cortelli P, Montagna P, Autilio-Gambetti L, Gajdusek DC, Lugaresi E (1994). A novel mechanism of phenotypic heterogeneity demonstrated by the effect of a polymorphism on a pathogenic mutation in the PRNP (prion protein gene). Mol Neurobiol.

[36] Pietrini V, Puoti G, Limido L, Rossi G, Di Fede G, Giaccone G, Mangieri M, Tedeschi F, Bondavalli A, Mancia D (2003). Creutzfeldt-Jakob disease with a novel extra-repeat insertional mutation in the PRNP gene. Neurology.

[37] Pocchiari M, Poleggi A, Principe S, Graziano S, Cardone F (2009). Genomic and post-genomic analyses of human prion diseases. Genome Med.

[38] Poleggi A, van der Lee S, Capellari S, Puopolo M, Ladogana A, De Pascali E, Lia D, Formato A, Bartoletti-Stella A, Parchi P et al (2018) Age at onset of genetic (E200K) and sporadic Creutzfeldt-Jakob diseases is modulated by the CYP4X1 gene. J Neurol Neurosurg Psychiatry. 10.1136/jnnp-2018-318756 10.1136/jnnp-2018-318756 30032116

[39] Prusiner SB (1998). The prion diseases. Brain Pathol.

[40] Puoti G, Bizzi A, Forloni G, Safar JG, Tagliavini F, Gambetti P (2012). Sporadic human prion diseases: molecular insights and diagnosis. Lancet Neurol.

[41] Puoti G, Di Fede G, Cotrufo R, Tucci C, Capuano G, Giaccone G, Tagliavini F (2004). Insertional mutation in the prion protein gene presenting with schizophrenia. Neurobiol Aging.

[42] Roeber S, Grasbon-Frodl EM, Windl O, Krebs B, Xiang W, Vollmert C, Illig T, Schröter A, Arzberger T, Weber P et al (2008) Evidence for a pathogenic role of different mutations at codon 188 of PRNP. PLoS One 3(5):e2147. 10.1371/journal.pone.0002147. 10.1371/journal.pone.0002147 PMC2366066 18478114

[43] Salvatore M, Genuardi M, Petraroli R, Masullo C, D'Alessandro M, Pocchiari M (1994). Polymorphisms of the prion protein gene in Italian patients with Creutzfeldt-Jakob disease. Hum Genet.

[44] Schätzl HM, Da Costa M, Taylor L, Cohen FE, Prusiner SB (1995). Prion protein gene variation among primates. J Mol Biol.

[45] Schmitz M, Dittmar K, Llorens F, Gelpi E, Ferrer I, Schulz-Schaeffer WJ, Zerr I (2017). Hereditary human prion diseases: an update. Mol Neurobiol.

[46] Sim N-L, Kumar P, Hu J, Henikoff S, Schneider G, Ng PC (2012). SIFT web server: predicting effects of amino acid substitutions on proteins. Nucleic Acids Res.

[47] Stone EA, Sidow A (2005). Physicochemical constraint violation by missense substitutions mediates impairment of protein function and disease severity. Genome Res.

[48] Tunnell E, Wollman R, Mallik S, Cortes CJ, Dearmond SJ, Mastrianni JA (2008). A novel PRNP-P105S mutation associated with atypical prion disease and a rare PrPSc conformation. Neurology.

[49] Will RG (2003). Acquired prion disease: iatrogenic CJD, variant CJD, kuru. Br Med Bull.

[50] Windl O, Dempster M, Estibeiro JP, Lathe R, de Silva R, Esmonde T, Will R, Springbett A, Campbell TA, Sidle KC (1996). Genetic basis of Creutzfeldt-Jakob disease in the United Kingdom: a systematic analysis of predisposing mutations and allelic variation in the PRNP gene. Hum Genet.

[51] Zerr I, Kallenberg K, Summers DM, Romero C, Taratuto A, Heinemann U, Breithaupt M, Varges D, Meissner B, Ladogana A (2009). Updated clinical diagnostic criteria for sporadic Creutzfeldt-Jakob disease. Brain.

[52] Zhang W, Jiao B, Xiao T, Pan C, Liu X, Zhou L, Tang B, Shen L (2016). Mutational analysis of PRNP in Alzheimer’s disease and frontotemporal dementia in China. Sci Rep.

