# Supplementary material for: Clinical and neuropathological phenotype associated with the novel V189I mutation in the prion protein gene
Source: Acta Neuropathol Commun. 2019 Jan 3;7:1. doi: 10.1186/s40478-018-0656-4 (PMC6317215; doi:10.1186/s40478-018-0656-4)
Supplement: Supplementary file 1 — Figure S1. Neuropathology of case 3. The neuropathological study on brain sample from case 3 showed findings overlapping those found in case 1 and 4. Severe neuronal loss and spongiform changes (a: frontal cortex, Haematoxylin-Eosin) associated with astrogliosis (b: frontal cortex, GFAP immunostaining) were observed in cerebral cortex. The pattern of PrPSc deposition was the same of cases 1 and 4, and consisted of diffuse, synaptic-like immunoreactivity (c: 3F4 immunostaining, frontal cortex). Similar changes were found in cerebellum: loss of Purkinje cells and spongiosis in the molecular layer (d: Haematoxylin-Eosin), diffuse astrogliosis in the granular layer (e: GFAP immunostaining) and finely granular PrP deposits in the molecular layer (f: 3F4 immunostaining). As in the other cases carrying the V189I mutation, coarse spots of PrP immunostaining were evident in the granular layer (f: 3F4 immunostaining). Scale bars: in (a) = 100 μm (a, b, d and f are the same magnification); in (c) = 50 μm (c and e are the same magnification). (PDF 2218 kb) [file 40478_2018_656_MOESM1_ESM.pdf]

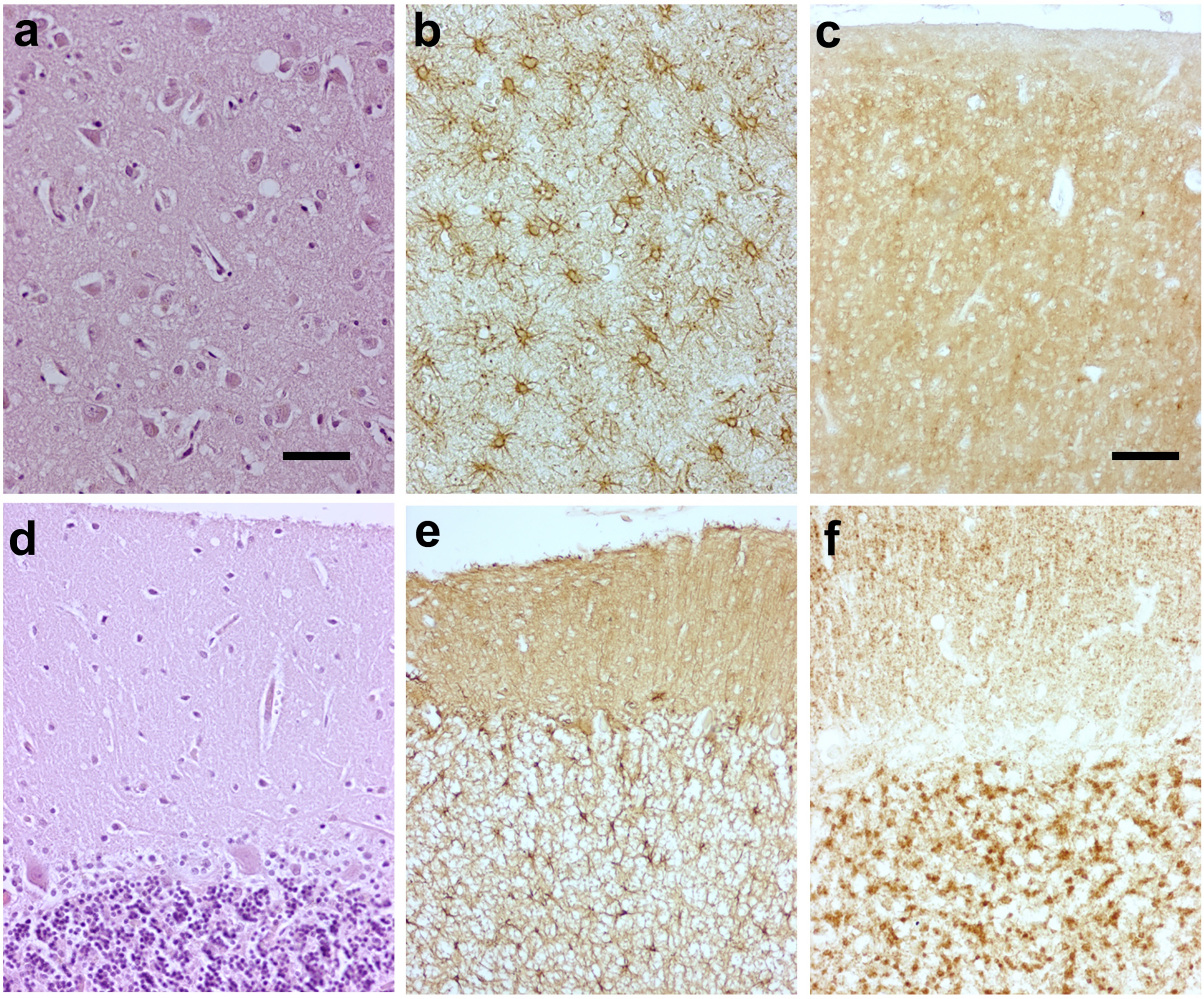

Fig. S1 Neuropathology of case 3. The neuropathologic study on brain sample from case 3 showed findings overlapping those found in case 1 and 4. Severe neuronal loss and spongiform changes (**a**: frontal cortex, Haematoxylin-Eosin) associated with astrogliosis (**b**: frontal cortex, GFAP immunostaining) were observed in cerebral cortex. The pattern of PrP^{Sc} deposition was the same of cases 1 and 4, and consisted of diffuse, synaptic-like immunoreactivity (**c**: 3F4 immunostaining, frontal cortex). Similar changes were found in cerebellum: loss of Purkinje cells and spongiosis in the molecular layer (**d**: Haematoxylin-Eosin), diffuse astrogliosis in the granular layer (**e**: GFAP immunostaining) and finely granular PrP deposits in the molecular layer (**f**: 3F4 immunostaining). As in the other cases carrying the V189I mutation, coarse spots of PrP immunostaining were evident in the granular layer (**f**: 3F4 immunostaining).

Scale bars: in (**a**) = 100 µm (**a**, **b**, **d** and **f** are the same magnification); in (**c**) = 50 µm (**c** and **e** are the same magnification).
